# Effects of irregular respiratory motion on the positioning accuracy of moving target with free breathing cone-beam computerized tomography

**DOI:** 10.4236/ijmpcero.2018.72015

**Published:** 2018-05-08

**Authors:** Xiang Li, Tianfang Li, Ellen Yorke, Gig Mageras, Xiaoli Tang, Maria Chan, Weijun Xiong, Marsha Reyngold, Richard Gewanter, Abraham Wu, John Cuaron, Margie Hunt

**Affiliations:** 1 Dept. of Medical Physics, Memorial Sloan Kettering Cancer Center, New York, NY; 2 Dept. of Radiation Oncology, Memorial Sloan Kettering Cancer Center, New York, NY

**Keywords:** Cone beam computerized tomography, respiratory motion effect

## Abstract

For positioning a moving target, a maximum intensity projection (MIP) or average intensity projection (AIP) image derived from 4DCT is often used as the reference image which is matched to free breathing cone-beam CT (FBCBCT) before treatment. This method can be highly accurate if the respiratory motion of the patient is stable. However, a patient’s breathing pattern is often irregular. The purpose of this study is to investigate the effects of irregular respiration on positioning accuracy for a moving target aligned with FBCBCT. Nine patients’ respiratory motion curves were selected to drive a Quasar motion phantom with one embedded cubic and two spherical targets. A 4DCT of the phantom was acquired on a CT scanner (Philips Brilliance 16) equipped with a Varian RPM system. The phase binned 4DCT images and the corresponding MIP and AIP images were transferred into Eclipse for analysis. FBCBCTs of the phantom driven by the same respiratory curves were also acquired on a Varian TrueBeam and fused such that both CBCT and MIP/AIP images share the same target zero positions. The sphere and cube volumes and centroid differences (alignment error) determined by MIP, AIP and FBCBCT images were calculated, respectively. Compared to the volume determined by MIP, the volumes of the cube, large sphere, and small sphere in AIP and FBCBCT images were smaller. The alignment errors for the cube, large sphere and small sphere with center to center matches between MIP and FBCBCT were 2.5 ± 1.8mm, 2.4±2.1 mm, and 3.8±2.8 mm, and the alignment errors between AIP and FBCBCT were 0.5±1.1mm, 0.3±0.8mm, and 1.8±2.0 mm, respectively. AIP images appear to be superior reference images to MIP images. However, irregular respiratory pattern could compromise the positioning accuracy, especially for smaller targets.

## 1. Introduction

On-board cone-beam CT (CBCT) plays an important role in image-guided radiotherapy and stereotactic radio-surgery. However, respiratory motion poses a particular challenge for aligning a moving target with free breathing CBCT (FBCBCT). Due to the slow scanning speed of the on-board CBCT relative to the respiratory motion, the acquired projection images of a moving target are comprised of a mixture of phases from different respiratory cycles, resulting in significant motion artifacts in the reconstructed FBCBCT images. In the clinic, rather than a normal Helical CT, the maximum intensity projection (MIP) images or average intensity projection (AIP) images derived from a 4D CT dataset have been suggested as reference images to give better alignment with FBCBCT [1–2]. Recent phantom studies have shown that the shape and centroid position of the objects agree with each other between MIP and FBCBCT images when the breathing motion follows a sinusoidal pattern [3], but the MIP image might overestimate the target volume compared with that determined by an on-board FBCBCT with irregular respiratory motion pattern and cause systematic positioning errors [4–6], and AIP image is suggested to be a better alternative. In these phantom studies, different respiratory motion amplitudes and different ratios of exhale and inhale duration (EIR) were simulated to describe the irregularity of the respiratory motion. However, for every cycle in each tested curve the amplitude, frequency and EIR were fixed. For a real patient’s breathing pattern, the respiratory motion amplitude, frequency and EIR can vary over the CBCT acquisition time, which may further deteriorate the target positioning accuracy. This study aims to quantify the effect of irregular respiratory motion on the positioning accuracy of a moving target with FBCBCT.

## 2. Materials and methods

### 2.1. Free breathing CBCT

It is important to realize what FBCBCT represents for moving targets. In clinic, filtered back-projection (FBP) reconstruction algorithm is usually used to reconstruct the CBCT images. Equation (1) describes the basic concept of FBP based CBCT reconstruction.

(1) $$I\left(x,y,z\right)=\int_{0}^{2\pi }BP\left(x,y,z,\beta \right)\{\left[w\left(x,y,z,\beta \right)P\left(a\left(x,y,z,\beta \right),b\left(x,y,x,\beta \right),\beta \right)\right]*g\left(a\right)\}d\beta$$

Where *I(x,y,z)* is the image intensities of the reconstructed CBCT image at a voxel (x, y, z) *and* P(a,b,β) is the corresponding projection (after logarithmic transformation) on the flat panel detector at the projection angle β, respectively. *w()* function is the pre-weighted geometric factor, and *g()* is a 1-D ramp filtering function. The function *BP()* stands for the back projection operation. More detailed descriptions of each function can be referred to the reference [7]. For the purpose of simplicity, we can rewrite the equation (1) as follows:

(2) $$I\left(x,y,z\right)=\int_{0}^{2\pi }\mathrm{FBP}(p\left(a\left(x,y,z,\beta \right),b\left(x,y,z,\beta \right),\beta \right)d\beta$$

For a moving target, the projection images can be sorted into *n* different time intervals within respiratory cycles, and the equation (2) can be rewritten as follows:

(3) $$I\left(x,y,z\right)=\sum_{i=0}^{n-1}\int_{0}^{2\pi }\mathrm{FBP}(p\left(a\left(x,y,z,\beta \right),b\left(x,y,z,\beta \right),\beta ,\beta \varepsilon i\right)d\beta$$

where *i* is the index of the respiratory phase *i*, ranging from 0 to *n-1*. The equation (3) can be further simplified as:

(4) $$I\left(x,y,z\right)=\sum_{i=0}^{n-1}w_{i}I_{i}\left(x,y,z\right))$$

where $I_{i}\left(x,y,x\right)$ is the reconstructed CBCT image at the *i*th respiratory phase. If the respiratory phases are sorted with equal time (?) interval within each respiratory phase, the weighting factors $w_{i}$ would be almost same for all phases. This means that the FBCBCT is approximately the same as the average intensity projection image of 4DCBCT images as seen in equation (5):

(5) FBCBCT≈AIP(phase binned4DCBCT)

Therefore, if a patient has the same perfectly reproducible respiratory motion pattern at treatment and simulation, theoretically the AIP of 4DCT should be an excellent representation of FBCBCT even if 4DCBCT is not available.

### 2.2. 4DCT and CBCT acquisition

**Figure 1:**
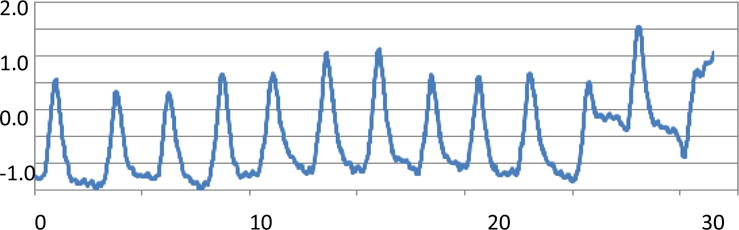
A respiratory motion curve with asymmetrical inhale and exhale duration, varying amplitude and frequency

**Fig 2:**
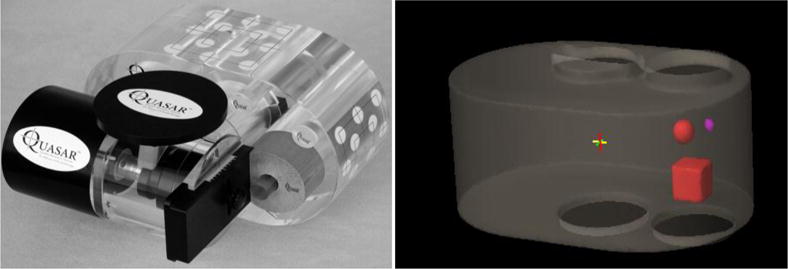
QUASAR respiratory motion phantom with embedded cubic and spherical objects

In reality, a patient’s respiratory motion is not always reproducible. The frequencies, amplitudes, ratios of exhale vs inhale duration, or even the baseline could change from time to time during CBCT image acquisition. One example is shown in Figure 1. If AIP of 4DCT images registered with the FBCBCT for target localization, the irregular respiratory motion might adversely affect the alignment accuracy. To quantify the effect, 9 clinical respiratory motion curves acquired with the Realtime Position Monitoring ™ system (Varian Medical Systems,…) were randomly selected with respiratory motion amplitudes ranging from 0.6 to 2.0cm, and the curves were used to drive a QUASAR motion phantom (Modus Medical Devices Inc., USA) during CT/CBCT scans. The phantom contains a 4DCT image insert which has three embedded image features: one cube with a width of 3cm and two spheres with diameters of 1cm and 2cm, respectively. Figure 2 shows a photo of the Quasar phantom used in this study (left) and a surface rendering image of the three image features (right).

Before each 4DCT acquisition, the motion phantom was calibrated at the amplitude position of zero and a 3D CT dataset was acquired at the amplitude of the 0cm location. In this study, all 3D and 4DCT images were acquired with a Philips Big Bore 16-slice CT simulator using the vendor-supplied retrospective helical 4DCT reconstruction software. The 4DCT protocol utilizes a 0.44 second rotation speed with a pitch of 0.065. The slice thickness of the reconstructed CT images was 2 mm with dimensions of 512×512 pixels. Phase binning was chosen to sort out the 4DCT images with 10-phase interval. All the CT images were imported into an Eclipse treatment planning system (Version 13.6, Varian Incorporation) for further analyses. In the Eclipse planning system, MIP and AIP images were created from the 4DCT images for each respiratory motion curve, and a setup plan was generated on the static 3D CT images acquired at the phantom amplitude position of 0 with an isocenter set at the center of the 4DCT image insert. A CBCT setup field was added into each plan for the CBCT acquisition on a treatment machine. In this study a Varian True Beam machine with a version of MR 2.5 was used to acquire the CBCT images in a half fan mode. Initially, the Quasar phantom placed on a True-Beam 6D Calypso couch was calibrated the same way as for the 4DCT simulation, and the phantom was set static at the amplitude of 0. CBCT was acquired and fused with the static 3D planning CT images. Based on the fusion result, 6D corrections were made to position the Quasar phantom so that the zero positions of the moving targets are the same between FBCBCT and 4DCT images. Afterwards, the same respiratory motion curves were used to drive the Quasar motion phantom, and a series of FBCBCT was taken. In this way the FBCBCT images were naturally registered with the corresponding 4DCT images for each respiratory curve.

### 2.3. Data analysis

**Figure 3:**
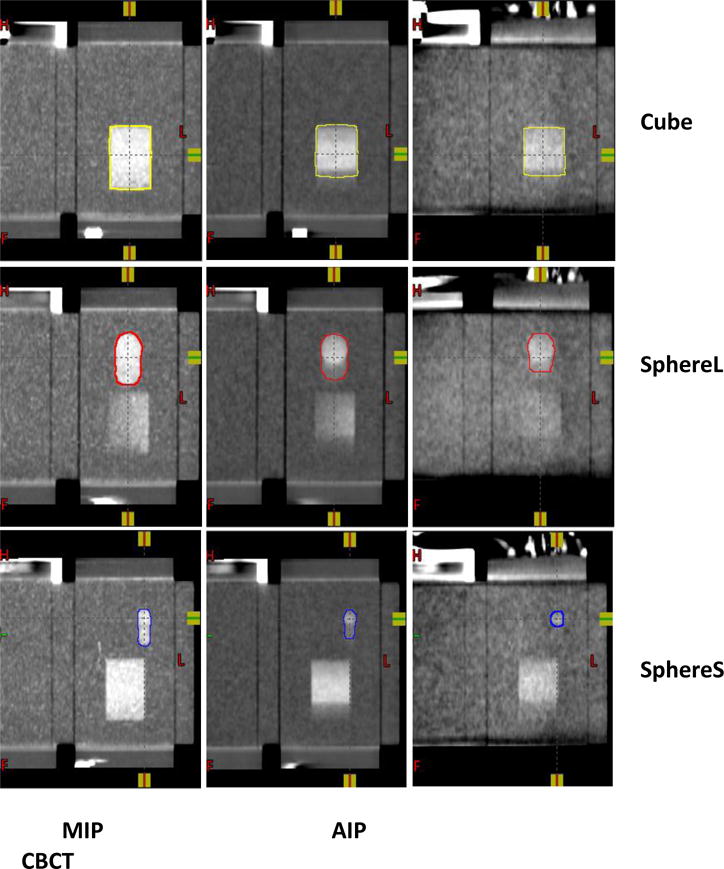
Targets contouring in MIP, AIP and CBCT images.

For each respiratory motion curve, MIP, AIP and FBCBCT were automatically registered after CBCT acquisition and were reviewed and analyzed in the Eclipse system. In this paper, target volume and alignment error with center to center match strategy were used to quantify the effect of irregular respiratory motion on FBCBCT images. Figure 3 shows the MIP, AIP and FBCBCT images for the respiratory motion curve in Figure 1. The mean intensity of the target and background in the CT scan is about 350 and 130 HU, respectively. First, the threshold of 180HU was used to automatically segment the targets from the background, and then a low-pass filter was adopted to smooth the contour for each target. The yellow, red and blue lines in Figure 3 indicate the contours of the cube, large sphere, and small sphere, respectively. The volume and the centroid of each target were recorded. Since the MIP and AIP images have already been registered with FBCBCT, any location difference of the centroid among MIP, AIP and FBCBCT would be due to alignment error if the target center to center match was used to locate the target on FBCBCT.

## 3. Results

### 3.1. Comparison of the tumor volume between MIP, AIP and FBCBCT image

**Figure4:**
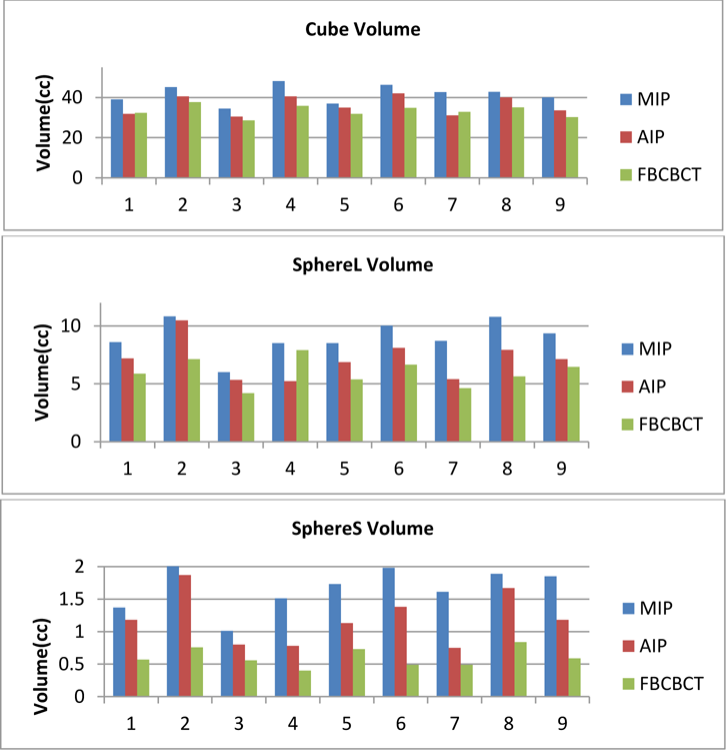
Target volume differences between MIP, AIP and FBCBCT

Figure 4 shows the target volumes determined by MIP, AIP and FBCBCT images for all nine respiratory curves. Compared to the ITV volumes determined by MIP images, the cube volumes were 12.7% ±7.0% and 20.1%±4.4% smaller on AIP and CBCT images; the larger sphere volumes were 13.8% ±7.9% and 34.2%±9.6% smaller on AIP and CBCT images; and the smaller sphere volumes were 15.2% ±13.1% and 64.0%±10.9% smaller on AIP and CBCT images. It can be clearly seen that the MIP images tend to overestimate the target volume determined by the FBCBCT image, and the AIP image is closer to the FBCBCT image but can also significantly overestimate the ITV volumes, especially for smaller targets. For example, in the bottom line of Figure 3 the small sphere volume shown in FBCBCT was less than half of the volume shown in the AIP image.

### 3.2. Alignment errors with center to center match between MIP vs FBCBCT and AIP vs FBCBCT

**Figure 5:**
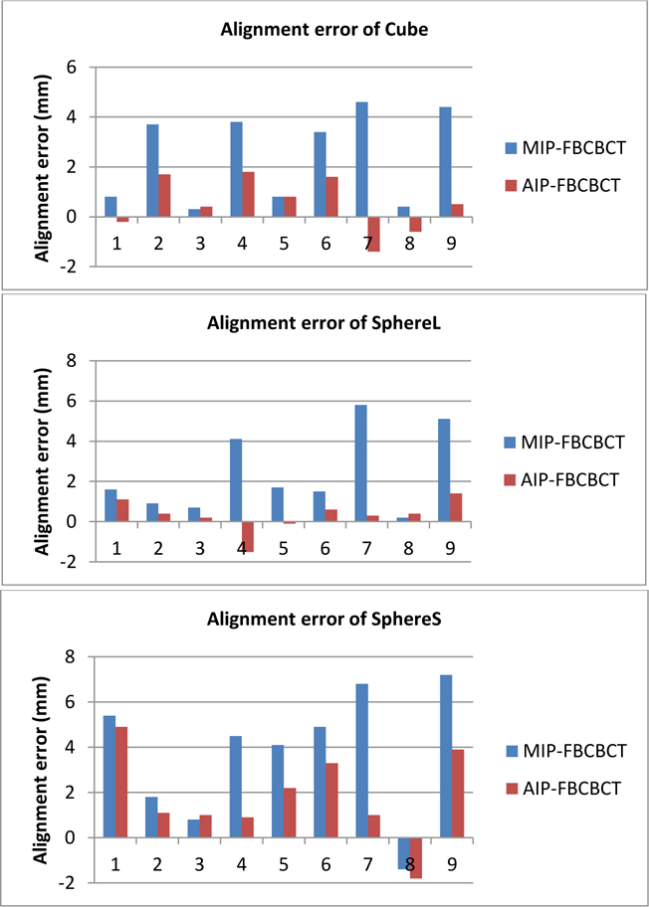
Alignment error with center-center match between MIP-FBCBCT and AIP-FBCBCT

As Quasar phantom is a 1-D respiratory motion platform, it only generates respiratory motion along the Superior Inferior (SI) direction, so the target centroid differences between MIP vs FBCBCT and AIP vs FBCBCT are mainly the displacements along the SI direction. Figure 5 shows alignment errors between MIP-FBCBCT and AIP-FBCBCT. For the cube and large sphere, the alignment errors with center to center match between MIP and FBCBCT were up to 6 mm with average errors of 2.5 ± 1.8mm and 2.4 ± 2.1mm, respectively. On the contrary, the maximum alignment errors were reduced to less than 2mm for center to center match between AIP and FBCBCT, and the average alignment errors of the cube and large sphere were 0.5 ± 1.1mm and 0.3 ± 0.8mm, respectively. For the smaller sphere, the alignment errors were 3.8 ± 2.8mm and 1.8 ± 2.0mm for MIP-FBCBCT and AIP-FBCBCT with center-center match, respectively.

## 4. Discussion

We noticed that the center to center match based on either MIP or AIP could lead to reasonable match accuracy if a patient has a sinusoid-like respiratory motion pattern in which the EIR was nearly 1. This is consistent with Wang’s results in the reference [3]. However, for the majority of thepatients, exhale duration is longer than the inhale duration [8]. In these cases using MIP image as a reference image could result in a large setup error when there is significant respiratory motion. On the contrary, the AIP image can still provide reasonable alignment accuracy. Therefore, AIP is recommended to serve as a reference image when matching with FBCBCT image for moving tumor treatments.

According to Equation 5 the characteristics of the moving lung tumor determined by FBCBCT image can be approximately estimated by the AIP image of 4DCT if the patient has a reproducible respiratory motion pattern between simulation and treatment. However, our results showed that sometimes even the AIP image fails to accurately predict the FBCBCT because there are some fundamental differences between AIPs derived from 4DCT and FBCBCT. First, FBCBCT is approximately the average intensity projection image of 4DCBCT image across 10 to 20 respiratory cycles for most of the patients, but a lung tumor with medium size is usually scanned within 1~3 respiratory cycles in 4DCT acquisition, especially with a multi-slice scanner. This means that AIP from 4DCT is more sensitive to irregular respiratory motion and may not be a good predictor if the tumor is scanned at a time when an irregular respiratory motion occurs, e.g., motion amplitude, frequency, or EIR are different from normal breathing cycles. Secondly, compared to 4DCT scan, due to the nature of large cone geometry, FBCBCT suffers increased noise level and scatter artifacts [9]. It is well known that scatter effect can significantly reduce the image contrast and cause streak artifacts. Because of the noise and scatter effects, some subtle features with very low image contrast that could be detected by AIP images might be buried in the FBCBCT image. For example, as seen in Figure 3, for the smaller sphere, part of the respiratory motion trace could be seen in AIP image, but totally disappeared on FBCBCT scan. Even though in general AIP is a better reference image than MIP image, there are still some uncertainties when aligning with FBCBT due to the irregular respiratory motion, especially for smaller targets. The current 5~7mm margin commonly adopted in clinic seems adequate and necessary to compensate the setup uncertainty due to irregular respiratory motion.

**Figure 6:**
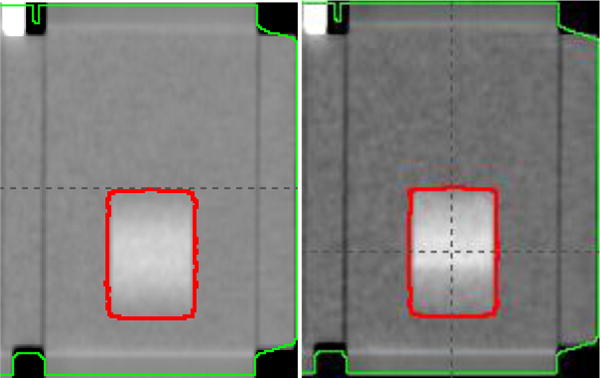
AIP images amplitude vs phase binning

It should also be pointed out that the AIP image should be only derived from phase binned 4DCT images. Philip Big Bore 4DCT software offers two type of binning methods: amplitude and phase binning [10]. Phase binning divides a respiratory cycle into ten phases with equal time intervals, and amplitude binning separates the breathing cycle according to the amplitude only and does not consider the temporal contribution. Therefore, phase binned 4DCT images carry the temporal information of the tumor respiratory pattern, but amplitude binned 4DCT does not. One common mistake is to use amplitude binned 4DCT to generate AIP image serving as a reference image, which would lead a large setup error. Figure 6 shows an example that was acquired given the respiratory motion curve shown in Figure 1. The red contour indicates the ITV volume. The left and right images are the AIP images derived from amplitude binning and phase binning 4DCT images, respectively. Since the patient has prolonged exhale duration, it is expected that in the AIP image there would be higher image intensity on the superior portion which corresponds to the exhale stage. This is the case for the AIP image derived from phase binned 4DCT, but is not true for the amplitude binned 4DCT images because it does not contain temporal information.

Ultimately, more accurate moving tumor alignment can be achieved with the help of 4DCBCT. There have been many research efforts in developing the 4DCBCT technique [11–13]. Currently Elekta Inc. has produced linear accelerators with 4DCBCT capability, and Varian Inc. will release their 4DCBCT function on True-Beam machine in the version of MR2.7. Compared to FBCBCT, 4DCBCT images offer a better image quality with fewer motion artifacts and sharper boundaries, and reveal the patient’s respiratory motion pattern within breathing cycle. Therefore, a user should be able to quantify the respiratory motion range and ensure that the tumor motion can be confined within the ITV volume during the treatment. To meet the clinical need for fast CBCT reconstruction, FBP like algorithms are generally used in the above 4DCBCT system. Due to the relatively small number of projections for each respiratory phase, the reconstructed 4DCBCT images usually suffer higher noise level and streak artifacts. Compressed sensing technique and GPU based programming have been investigated to further improve the 4DCBCT image quality and reconstruction speed [14]. However, despite of all those efforts, there are still significant numbers of linear accelerators without 4DCBCT functionality running in clinic. When patients with moving tumors are treated on these machines, special attentions should be made to ensure proper target alignment. Compared to MIP image, AIP image derived from 4DCT images appear to be the superior reference image. Other alternative respiratory motion suppression methods, such as compression belt [15] or deep inspiration breath hold (DIBH) [16] can also be used to minimize the respiratory motion effect on FBCBCT images.

## 5. Conclusions

The characteristics of a moving target determined by FBCBCT imaging are highly dependent on the patient’s specific respiratory motion pattern. Even though AIP image derived from 4DCT appears to be the superior reference image to MIP, irregular respiratory motion can still compromise the alignment accuracy when target center to center match strategy is adopted, and adequate PTV margin is necessary to ensure adequate PTV coverage.
